# Patient and provider perceptions of the relationship between alcohol use and TB and readiness for treatment: a qualitative study in South Africa

**DOI:** 10.1186/s12889-024-19570-y

**Published:** 2024-08-14

**Authors:** Suchitra Kulkarni, Sarah E. Weber, Chané Buys, Tersius Lambrechts, Bronwyn Myers, Mari-Lynn Drainoni, Karen R. Jacobson, Danie Theron, Tara Carney

**Affiliations:** 1 Section of Infectious Diseases, Department of Medicine, Boston University Chobanian Avedisian School of Medicine and Boston Medical Center, Boston, Massachusetts United States of America; 2 Mental Health, Alcohol, Substance Use and Tobacco Research Unit, South African Medical Research Council, Cape Town, South Africa; 3 Department of Psychiatry and Mental Health, University of Cape Town, Cape Town, South Africa; 4 Curtain enAble Institute, Faculty of Health Sciences, Curtain University, Perth, Australia; 5 Department of Health Law, Policy and Management, Boston University School of Public Health, Boston, Massachusetts USA; 6 Brewelskloof Hospital, Worcester, South Africa

**Keywords:** Alcohol, Tuberculosis, South Africa, Healthcare systems, Behavior change

## Abstract

**Background:**

Unhealthy alcohol use is widespread in South Africa and has been linked to tuberculosis (TB) disease and poor treatment outcomes. This study used qualitative methods to explore the relationship between TB and alcohol use during TB treatment.

**Methods:**

Focus group discussions (FGDs) were conducted with 34 participants who had previous or current drug-susceptible TB and self-reported current alcohol use. Eight interviews were conducted with healthcare workers who provide TB services in Worcester, South Africa.

**Results:**

In this rural setting, heavy episodic drinking is normalized and perceived to be related to TB transmission and decreased adherence to TB medication. Both healthcare workers and FGD participants recommended the introduction of universal screening, brief interventions, and referral to specialized care for unhealthy alcohol use. However, participants also discussed barriers to the provision of these services, such as limited awareness of the link between alcohol and TB. Healthcare workers also specified resource constraints, while FGD participants or patients mentioned widespread stigma towards people with alcohol concerns. Both FGD participants and health providers would benefit from education on the relationship between TB and unhealthy alcohol use and had specific recommendations about interventions for alcohol use reduction. Healthcare workers also suggested that community health worker-delivered interventions could support access to and engagement in both TB and alcohol-related services.

**Conclusion:**

Findings support strengthening accessible, specialized services for the identification and provision of interventions and psychosocial services for unhealthy alcohol use among those with TB.

**Supplementary Information:**

The online version contains supplementary material available at 10.1186/s12889-024-19570-y.

## Background

Tuberculosis (TB) is the leading infectious disease-related cause of death globally [1]. Unhealthy alcohol use is a leading risk factor for acquiring TB infection and developing TB disease [2]. Among those diagnosed with TB disease, the prevalence of unhealthy alcohol use ranges from 15 to 70% [3–7]. Additionally, unhealthy alcohol use has been associated with mortality and loss to follow up in people living with TB [8–12], especially in the first few months after TB treatment completion [13]. In South Africa, a low- to middle-income country (LMIC) with a high TB disease burden and prevalence of unhealthy alcohol use, poor TB treatment outcomes have been documented for people with alcohol concerns [14–16]. These findings point to the need to uniformly screen patients with TB diagnosis for unhealthy alcohol use so that patients who might benefit from alcohol reduction interventions are identified and appropriately supported.

There is a growing commitment to integrating screening, brief interventions and referral to treatment (SBIRT) for unhealthy alcohol use into primary health care services in South Africa, including for chronic disease management such as HIV [14, 17] and also for emergency services [18]. However, SBIRT for unhealthy alcohol use is not routinely offered within TB services unless these services are co-located with HIV treatment. Currently, people with TB disease who disclose alcohol use are advised to stop drinking to avoid hepatotoxicity with TB medications and sometimes referred to mental health services for further assessment and potentially to specialist substance use disorder treatment facilities.

It is therefore not surprising that if people with TB disease seek healthcare, they are often reluctant to discuss alcohol use with their providers due to stigma and discrimination concerns. For example, in a recent South African study, only 37% of TB patients with an alcohol-related problem reported that they would discuss their alcohol use with healthcare clinic staff [19]. Interventions that address stigma and other barriers to accessing services for unhealthy alcohol use in people with TB disease are needed to improve their uptake of healthcare services and ultimately their health outcomes.

People with TB who use alcohol may experience multiple levels of stigma which can affect their access to healthcare [20]. Studies have only recently begun to look at stigma among individuals with unhealthy alcohol use and other health conditions, namely HIV, in South Africa [20, 21]. Limited research exists examining the impact of stigma in the context of simultaneous alcohol use and TB disease and how this may impact health [22, 23].

This study aimed to explore patient and health provider perceptions of the relationship between unhealthy alcohol use and TB disease using qualitative research methods, through the description of the experience of alcohol use during TB treatment, services provided for unhealthy alcohol use among those with TB disease, as well as recommendations for screening, intervention and referral to more specialized treatment.

## Methods

### Study design and setting

This qualitative sub-study is part of the Tuberculosis Treatment and Alcohol Use Study (TRUST). TRUST is a longitudinal, repeated measures, prospective cohort study that aims to examine the associations between alcohol use and drug-susceptible TB treatment outcomes in adults (age > 15 years) [24]. The study is conducted in Worcester, South Africa, a rural community with a high burden of TB and unhealthy alcohol use. This qualitative sub-study was conducted to explore the experiences of patients diagnosed with TB who self-reported alcohol use, as well as those of healthcare professionals working in TB services. The study is presented according to Consolidated Criteria for Reporting Qualitative Research (COREQ) guidance [25].

### Participants and recruitment procedures

Five focus group discussions (FGDs) were conducted with a subset of TRUST participants who had been receiving treatment for TB at the healthcare clinic. Of the five FGDs, three were held with men (*n =* 24) and two with women (*n =* 10); each group comprised four to eight participants. All participants identified as being of mixed race ancestry (referred to as Coloured in South Africa). Eligibility criteria included previous or current participation in TRUST, TB treatment completion, and self-reported current alcohol use at last study visit. Eligible participants from TRUST were randomly contacted to ascertain interest in and availability to participate in the sub-study. If an eligible participant was unavailable, the next individual on the list was contacted.

In-depth interviews (IDIs) were conducted with eight healthcare workers, who served as key informants (KIs). KIs were nurses who were purposively selected from the local hospitals and health care clinics run by the Western Cape Department of Health and had experience in working with patients with TB.

### Procedures

FGDs and IDIs were conducted between December 2021 and May 2022. They were facilitated by a male with a Masters-level education (in psychology) and supported by a female staff member who both spoke Afrikaans (predominant language spoken) and English. Neither facilitator was involved in the parent study and therefore were unknown to study participants. On the day of study activities, participants were rescreened to confirm eligibility, and facilitators outlined the study expectations and processes, obtained written informed consent, and collected sociodemographic information.

FGDs took place in a private room at a community hall. The facilitators followed a semi-structured guide comprised of open-ended questions and additional probes to enquire about the impact of unhealthy alcohol use on TB. Questions examined the relationship between alcohol and TB in the community and the effect that alcohol has on engagement in TB treatment. FGDs were audio-recorded with participants’ consent and lasted up to 90 min. Participants were provided with transportation as needed, refreshments, and a grocery voucher.

IDIs took place in a private office at either the study site or the participant’s place of work. Interviews followed a semi-structured guide with opening questions and follow-up probes to prompt KIs’ perceptions about alcohol use among individuals diagnosed with TB. Questions covered topics such as unhealthy alcohol use among TB patients, screening procedures for alcohol, services available for TB patients within and outside the healthcare setting, and barriers to treatment including stigma towards TB patients with unhealthy alcohol use. Interviews were audio-recorded and lasted up to 60 min.

The South African Medical Research Council (SAMRC) Ethics Committee (#EC011-5/2016) and the Institutional Review Board (IRB) of Boston Medical Center (BMC)/Boston University Medical Campus (#H-34970) provided ethics approval for the current study.

### Data analysis

Audio recordings were transcribed verbatim prior to data analysis, then transcriptions were read for emergent themes (familiarization with the data), with a thematic analysis approach used to conduct the data analysis [26]. The first two KI interviews and FGDs were coded independently by two independent Masters-level coders, and then two senior, PhD-level authors reviewed the codes (TC and MLD) generated to resolve any coding discrepancies. Both senior authors have experience in qualitative research methods, substance use and infectious disease with TC also being experienced in conducting research in this particular setting. The remaining transcripts were then coded, and once coding was finalized, themes were created. NVivo software was used to manage the qualitative data and high levels of agreement between the two coders were present.

## Results

### Participants

Among the 34 FGD participants, the average age was 40.8 years (SD = 5.9) and 71% were male. All of the KIs were nurses with an average of 9.7 years (SD = 9.6) of experience providing TB care. Most (89%) KI participants were female with an average age of 45.1 years (SD = 8.4) (Table 1).

**Table 1:** Study participant demographics

	In-depth Interview Participants (*N* = 8)	Focus Group Participants (*N* = 34)
Age in years, mean (SD)	45.13 (8.38)	40.79 (5.87)
Gender		
Male	1 (11%)	24 (71%)
Female	7 (89%)	10 (29%)
Language		
Afrikaans	7 (89%)	34 (100%)
English	1 (11%)	0 (0%)
Profession		
TB Nurse	6 (75%)	
General Nurse	1 (12.5%)	
Nurse Practitioner	1 (12.5%)	
Years in Services, mean (SD)	9.71 (9.61)	

Four major themes were identified: (1) normative community alcohol use despite risk for TB transmission; (2) differing patterns of alcohol use and motivations to change use during TB treatment; (3) identification and description of great need for alcohol-related services for people with TB disease; (4) different barriers to the uptake of existing alcohol services among healthcare workers and patients with TB disease (Table 2). These themes and related subthemes are described below and illustrated with quotes from participants.

**Table 2:** Study themes and subthemes

*Theme 1: Normative community alcohol use despite risk for TB transmission*
Subtheme 1: Alcohol use for social inclusion
Subtheme 2: Widespread availability of alcohol
*Theme 2: Differing patterns of alcohol use and motivation to change use during TB treatment*
Subtheme 1: Reduction in alcohol use during TB treatment
Subtheme 2: Continued alcohol use following TB diagnosis
*Theme 3: Identification and description of great need for alcohol-related services for people with TB disease*
Subtheme 1: Gaps in identifying alcohol related problems in TB healthcare
Subtheme 2: Gaps in intervention services for alcohol-related problems in TB healthcare
Subtheme 3: Suggested content of intervention services
Subtheme 4: Participant recommendations for intervention staff
Subtheme 5: Linkage to specialized care for alcohol use
*Theme 4: Difference in barriers to uptake of alcohol services among healthcare workers and patients with TB disease*
Subtheme 1: Individual motivation to change
Subtheme 2: Structural barriers to accessing services
Subtheme 3: Negative treatment by healthcare workers
Subtheme 4: Community-level barriers to healthcare

### Normative community alcohol use despite risk for TB transmission

#### Alcohol use for social inclusion

The majority of participants reported that heavy episodic alcohol use was normalized in the community. FGD participants described how in their view, drinking alcohol assists with social and community inclusion, mentioning that individuals would feel excluded and “alone” should they refrain from drinking in social situations. Some participants mentioned that this desire for social inclusion through “drinking from the same glass” supersedes concerns about TB transmission:

*“Three out of four times*,* you hear the cough. You don’t know where that cough is coming from. You aren’t used to the cough. But you know that person with you is a drinker. He coughs dangerously. But then you are also a drinker*,* then you just drink. But you don’t know*,* that illness is sitting in that glass. You drink in that sickness in just a drop in that glass.”* (Male Participant, FGD5).

KI participants also discussed the use of alcohol for social inclusion occuring within relationships and the deleterious effects of couples drinking together, including violence, jealousy and child neglect:

*“We have this culture of drinking*,* especially over weekends or believing that when there’s a salary or a weekend*,* then there should be drinking. So*,* there’s that hostile type of culture of drinking and socialising on weekends…so that’s why there’s been so much alcohol abuse*,* in people with TB…”* (Key Informant #3).

#### Widespread availability of alcohol

The majority of FGD participants and one KI participant described alcohol being widely available in their communities. A number of FGD participants spoke about the close proximity of alcohol outlets and lack of regulation around alcohol purchase, and one participant suggested that among people diagnosed with TB, alcohol is even more readily accessible as they receive social grants from the national government while on treatment, which in turn encouraged others to drink with them as it is known that they have money:

*“I have money for 6 months*,* while I have TB. Now who is going to reject when I have money. They’re going to say*,* come in*,* have a drink…if I knock at the shebeen [tavern]*,* they’re going to open up immediately because I have money.”* (Male Participant, FGD3).

### Differing patterns of alcohol use and motivations to change use during TB treatment

Participants expressed differing views about alcohol use during TB treatment, including how alcohol use may change in response to TB treatment initiation.

#### Reduction in alcohol use during TB treatment

A number of both FGD participants and KIs mentioned that in their experience, alcohol use changed during TB treatment. Some participants discussed a reduction in overall alcohol use frequency during TB treatment, with the exception of continued weekend drinking. Others expressed that there was often complete abstinence from alcohol, which continued with individuals “quitting completely” once treatment was complete. However, other FGD participants discussed that alcohol use resumed once TB-related symptoms subsided or treatment was complete:

*“It was my first time with TB. I began to take my tablets. I didn’t drink or smoke after that. After three months*,* I drank again but not with the tablets and maybe not on the same day.”* (Male Participant, FGD3).

There was a general consensus that initial alcohol use reduction during TB treatment was due to a number of reasons, including the belief that alcohol use during TB treatment intensifies symptoms and side effects when consuming alcohol with TB medications, such as feeling weak or nauseous. One participant discussed how alcohol exacerbated his TB symptoms:

*“I was very sick. I nearly died. I can’t believe I’m still alive. I say that the alcohol attacks the lungs*,* your life…I was nearly in the ground but I made it*,* man.”* (Male Participant, FGD3).

Both KIs and FGD participants discussed the perception that alcohol use during TB treatment reduces medication efficacy or “cancels out” TB medication, and is therefore detrimental to one’s health and ability to recover from TB. Again, both types of participants described how the effect of alcohol in exacerbating TB symptoms often leads to temporarily stopping alcohol use until TB symptoms subside:

*“…once they’ve been diagnosed then… then they promise not to drink again*,* but once they are on the medication for a few months…then the pattern just repeats itself and then they just use again*,* which then leads to the fact that they default and do not come again*,* because they feel better on the pills*,* they carry on with the alcohol.”* (Key Informant #5).

Several FGD participants noted changes in alcohol consumption to minimize overlapping TB medication and alcohol exposure. They spoke of delaying alcohol intake by a few hours after taking TB medication, sometimes following advice from healthcare clinic staff:

*“A lot of people say that if you drank your pills at eight o’clock then you can start drinking at one o’clock*,* because by then the pills are out of your body*,* such things are said*,* you hear such things*,* such things were told to me*,* that’s why I drank.”* (Female Participant, FGD2).

#### Continued alcohol use following TB diagnosis

Participants mentioned a number of reasons for persistent alcohol use during TB treatment, including alcohol being part of one’s lifestyle and “cravings”. Furthermore, FGD participants mentioned misperceptions about TB transmission and alcohol’s role in TB transmission:

*A lot of people don’t know that it’s a curable illness. A lot of people don’t know that it is spread through coughing or sputum or that it’s in the air.”* (Female Participant, FGD2).

*“I believe that whiskey*,* a strong drink*,* doesn’t affect TB. As I understand the thing*,* it is a strong alcohol and it burns everything inside. The same with the COVID-19*,* they say you mustn’t drink. But I think the best drink is the alcohol. Alcohol keeps the colds away.”* (Male Participant, FGD3).

One KI confirmed that TB patients continue to drink due to the prioritization of alcohol use, especially on weekends which in her experience, negatively affected treatment outcome:

*“Then you will find out: ‘Weekends we don’t take because weekends we drink.’ And those who have finished their pills … I mean they have finished six months*,* then the sputum is still positive. You ask her: ‘Sister I drank. I don’t have time for pills’”* (Key Informant #4).

### Identification and description of great need for alcohol-related services for people with TB disease

#### Gaps in identifying alcohol-related problems in TB healthcare

FGD participants and KIs both highlighted that inconsistent screening exists for unhealthy alcohol use among patients diagnosed with TB disease. Reasons that were provided included health care systems being overburdened, not enough time during patients’ visits to screen, and lack of training on appropriate and validated screening tools. FGD participants felt that screening for alcohol use should be conducted at every healthcare visit to build relationships and promote disclosure of alcohol use. Contrary to this, KIs discussed only screening individuals who *appeared* to present with an alcohol use problem, which was usually indicated by individuals being intoxicated during the healthcare visit, smelling like alcohol, or presenting with weight loss since their previous visit, but that this was also dependent on if time was available:

*When you’re under pressure it can happen that the patient screening can be overlooked*,* which is not effective.” (Key Informant #1)*.

#### Gaps in intervention services for alcohol-related problems in TB healthcare

When discussing existing intervention gaps, many participants cited a current lack of accurate knowledge among community members about alcohol use and potential harms, including the effect on TB disease, as well as TB disease in general. Healthcare worker participants identified that while nurses and other key clinic staff try to relay information during their visits, given time restraints, it would be helpful for another individual or lay counsellor to provide more in-depth interventions that support reductions in alcohol use. Some FGD participants reported that healthcare clinic staff generally provide patients with TB disease with some information about alcohol use and its interactions with TB treatment at the clinic, but others said that this was not common practice:

*“All that I ask*,* is to give people better information about TB please*,* please if they can handle people better and things can go better*,* and that people will want to complete their treatment…and everything will be better.”* (Female Participant, FGD2).

*“So*,* we do not always have that time to sit and talk to the patient. Because we help in the consultation*,* we can stress the importance of treatment. It would be great if we had a person that could spend more time with patients to educate them.”* (Key Informant #1).

#### Suggested content of intervention services

Both FGD and KI participants highlighted the importance of educating individuals with alcohol use about TB, including raising awareness about the effects of alcohol on TB treatment through information sharing at open days at health clinics:

*“People can get more information about TB*,* find out more about curable illnesses*,* how it is spread and support people so that they can complete their treatment*,* such small things.”* (Female Participant, FGD5).

*“If we have open days providing people with information on TB we educate them against TB*,* the conditions of the disease and [how] it presents itself in the community.”* (Key Informant #1).

In addition to providing information, participants discussed the need for other psychosocial services that may underpin alcohol concerns among individuals with unhealthy alcohol use who also have TB disease, such as skills development and work training:

*“Maybe we can think about college*,* education*,* skills training and the government gives money for it. So if the people just have something … drive them*,* I think they will act more responsibly in that sense – use their medication*,* how to handle the disease and the misuse of alcohol.”* (Male Participant, FGD5).

#### Participant recommendations around intervention staff

FGD participants also recommended training community health workers (CHWs) to deliver brief alcohol harm reduction interventions and provide additional social support for medication adherence and alcohol behavior change. They noted the importance of non-judgemental communication between CHWs and patients. With the correct training, KIs agreed that CHWs who provide directly observed therapy (DOTS) were optimally placed to deliver these interventions as they interact with patients for TB treatment on an almost daily basis and can develop relationships:

*“Because they see them every day and communicate with them every day*,* the patient is going to build a relationship over time and gain trust in the DOTS worker so when they are*,* I think*,* they’re at that level where they can both trust each other.* (Key Informant #3)

In addition, FGD participants in particular felt strongly that services such as peer support groups that offer social support for alcohol behavior change and TB treatment adherence would be helpful. They spoke about improving motivation to change behavior through hearing other stories of success and learning from individuals who had successfully completed TB treatment and reduced or discontinued their use of alcohol:

*“We must help each other. As we said now*,* go to the support groups. That what you have here is very good. If I could have just one a month*,* it would be very good. I think we will get bigger. We must initiate a plan of action like it. Yes*,* it can help.”* (Male Participant, FGD6).

#### Linkage to specialized care for alcohol use

Most FGD participants were not familiar with services that addressed both TB and alcohol use issues. They were also only aware of a limited number of services for individuals presenting with alcohol use disorders in the community or nearby, despite beliefs that treatment facilities provided useful services if individuals were motivated to change behavior:

*“If he wants to rehabilitate himself. If he doesn’t want to help himself*,* then he will give up.” *(Male Participant, FGD5).

Key stakeholders provided information on the process of linkage or referral to specialized substance use disorder treatment according to their experiences, usually directly after a positive screening for unhealthy alcohol use. Clinic social workers were most often responsible for facilitating the linkage, and although the aim is to provide patient-centered healthcare, waiting times were cited as a common reason for lack of uptake:

*“We do have an adequate referral system*,* what I think is perhaps lacking in our system is that our social worker is also very busy*,* and she then has to arrange appointments*,* which takes a very long time. Uhm*,* she can only see the patient in a few weeks’ time.”* (Key Informant #3).

### Different barriers to uptake of existing alcohol services among healthcare workers and patients with TB

#### Individual motivation to change

FGD participants believed that healthcare staff checking in regularly can provide motivation to continue TB treatment and discontinue alcohol use, but generally felt that this support was not present which led to a lack of motivation for change in alcohol use behavior:

*“That’s why people need lots of support to raise that willpower. We don’t have much support.” (Male Participant*,* FGD3)*.

#### Structural barriers to accessing services

According to both patient and healthcare worker participants, barriers to alcohol service uptake included structural factors such as limited financial resources, lack of transportation and long waiting times when patients arrive at their appointment at healthcare clinics:

*“And this is the thing*,* you must pay to go there and that’s the reason why a lot of people probably don’t go. Because a lot of people don’t have the money to pay for…to apply.”* (Female Participant, FGD2).

#### Negative treatment by healthcare workers

FGD participants explained that they experienced stigma from healthcare professionals, and they described how nurses sometimes treated patients in a negative, judgmental manner, especially those who indicated alcohol use or were not adhering to their treatment regimen.

*“By us*,* the way I’ve seen it when the next person who is an alcohol user gets tended to*,* they are treated badly. The nurse would scold or walk away. She won’t help you*,* but yet she just treated me kindly right before then. Suddenly her mood would change.”* (Male Participant, FGD1).

FGD participants said that these experiences of stigmatizing behavior led to patients feeling demotivated to continue their TB treatment, and created additional barriers for patients to return to healthcare clinics for appointments and continue to look after their health.

*“It does*,* yes. They are going to feel like*,* why should I use my treatment further or why should I go to the hospital daily or monthly where the sister treats me like I am a type of what can I say…”* (Female Participant, FGD2).

#### Community-level barriers to healthcare

FGD participants also felt that lack of education about TB and alcohol use contributed to differential treatment of individuals diagnosed with TB within their social group and by the broader community, as they described a complete cut-off in contact and individuals feeling that they are “not welcome” or isolated:

*“Yes*,* a TB patient generally becomes stigmatized and that is also one of the reasons why they don’t simply go and seek healthcare for TB*,* because they know that people are going to judge them and that when they return to their homes or to their area… ‘You were in the queue for TB’*,* ‘You were such and such’ so*,* obviously there is*,* yes. So*,* one can work on that.”* (Key Informant #9).

The fear and expectation of judgment and gossip from friends, family and other community members, and the expectation of being stigmatized was a major reason for delays in health seeking behavior or not seeking healthcare at all:

*“Or you are going to be afraid to fetch your pills from the clinic. You are afraid your neighbour will see you because you are going to wait long at the clinic*,* because people will talk.”* (Female Participant, FGD2).

## Discussion

In this study, we explored perceptions of unhealthy alcohol use and TB disease among healthcare professionals and individuals with self-reported alcohol use who had previously been treated for TB disease. Findings indicated that alcohol was widely used and associated with a desire for social inclusion within a community where alcohol use is widely available. Alcohol use during TB treatment varied, and was also perceived to increase the risk of TB transmission and reduce adherence to TB medication by some participants. Finally, gaps and barriers to utilizing existing available services for unhealthy alcohol use were discussed, as well as suggestions on how to improve these services and facilitate access for people who have TB and unhealthy alcohol use.

Participants felt there were challenges with reducing alcohol use permanently. Reductions in alcohol use were mainly reported as temporary until TB symptoms subsided, despite views that use contributed to negative TB outcomes such as treatment failure. Participants also cited possible behavioral reasons as to why this exists, especially among those with high levels of alcohol consumption as has been found in existing literature [11, 27, 28]. This was within a setting where heavy alcohol use is a cultural norm, similarly to other studies conducted in this region [29, 30]. However, this study found that among this sample of people with TB, social inclusion also influenced continued group alcohol use despite TB diagnoses.

In addition, study findings indicated a number of misperceptions among participants around alcohol use and TB transmission, including among healthcare workers. For example, sharing alcohol containers was often mentioned as a key mode of transmission, but there was considerably less discussion about coughing and the spread of airborne droplets, which has widely been identified as the key driver of transmission [1]. Similar misperceptions have been found in other South African studies, such as spreading TB by sharing utensils [31], cold air and environments that are dusty [32], unclean air and water, and no knowledge of transmission agents at all reported in one study [33]. Lack of knowledge about TB transmission pathways may delay health-seeking behavior and affect treatment adherence [34], which can have long-lasting impacts on TB disease progression.

Another misperception that emerged is confusion regarding when people should be referred to specialist treatment for alcohol as opposed to receiving a brief intervention, which healthcare workers can provide. Brief interventions have been identified as effective for reducing alcohol use in healthcare settings for a number of years [35] but limited studies have focused on people diagnosed with TB disease [36].

Our results indicate that it is important to engage affected individuals in *both* health services and alcohol use disorder treatment. Unfortunately, structural barriers to accessing specialist services for alcohol use disorder and alcohol services in primary care clinics remain largely similar to those reported by other studies conducted in South Africa [37, 38]. Participants recommended using CHWs such as DOTs workers to overcome these structural barriers to care, by training these workers to provide brief alcohol reduction interventions. This “task-sharing” model of care has been extensively trialed in HIV services to address substance use disorder and mental health problems [39–41] and recently piloted for people with TB disease, including multi-drug resistant TB, and alcohol use disorder [36, 42].

A key barrier to both the identification and treatment of TB and unhealthy alcohol use as mentioned particularly by paticipants who were part of the FGDs was that the fear of, and actual experience of, perceived judgment affected their decisions to utilize healthcare services or disclose TB to peers and family. These findings are similar to previous research which found that widespread stigma is experienced by people living with TB in South Africa [43, 44] and is a major challenge to service utilization. Recent local studies conducted with patients [21] found that stigma is indeed experienced at multiple levels for people with unhealthy alcohol use and is exacerbated if individuals are living with HIV, which often co-occurs with TB in this setting. Future studies could explore the types of stigma experienced among those diagnosed with TB disease who also present with unhealthy alcohol use, and how to address the negative treatment of healthcare patients.

### Strengths and limitations

This study has both strengths and limitations. A strength of this study was that it included perspectives from *both* health care providers and people diagnosed with TB who use alcohol. However, as with any qualitative study, our findings may not be generalizable due to small sample size, which is expected. Our sample size was especially impacted by COVID-19 restrictions around social distancing for groups. Secondly, almost all of the KIs were female. Although this is representative of the gender ratio of nurses in healthcare settings in the Western Cape, there were no male perspectives included for healthcare workers. Conversely, a study strength is that for FGDs, participants who self-identified both as male or female were included to provide balanced gender responses.

## Conclusion

The study showed that in a high-burden TB setting, there were a number of reasons for persistent unhealthy alcohol use and the need for additional services integrated into care to address both TB disease and alcohol. A need for uniform screening for alcohol use in the healthcare setting emerged from this study, as well as the potential delivery of intervention services by trained CHWs. Since there were a number of misperceptions about TB in general, as well as the association between alcohol use and TB disease, education would be useful for patients, the community and those who deliver services to them. Overall, the suggestion from these findings is to strengthen services to allow for the provision of accessible, specialised services for alcohol among those with TB in a non-judgemental manner, including a clear referral pathway for patients identified as having a potential alcohol use disorder.

### Electronic supplementary material

Below is the link to the electronic supplementary material.

Supplementary Material 1

Supplementary Material 2

## Data Availability

Data cannot be shared publicly because of participant confidentiality concerns. The Institutional Review Boards at the SAMRC, BMC/Boston University Medical Campus did not approve data sharing activities for this research study. De-identified data are available upon request to the respective Institutional Review Boards at adri.labuschagne@mrc.ac.za or medirb@bu.edu.
