# Supplementary material for: Patient and provider perceptions of the relationship between alcohol use and TB and readiness for treatment: a qualitative study in South Africa
Source: BMC Public Health. 2024 Aug 14;24:2216. doi: 10.1186/s12889-024-19570-y (PMC11325746; doi:10.1186/s12889-024-19570-y)
Supplement: Supplementary file 1 — Supplementary Material 1 [file 12889_2024_19570_MOESM1_ESM.pdf]

Participant Focus Group Guide

We are interested in alcohol use among individuals that have been diagnosed with TB, and how this may affect them. We will also ask you a few questions about the kinds of healthcare and other services that are available for these individuals, and what you think are some of their needs may be, as well as your thoughts about the impact of the COVID-19 pandemic on people's wellbeing. During this group, we would like to hear about your experiences and opinions. While we are asking you about people who use alcohol and have TB that you know or in your community, it is up to you if you would like to share your own personal information. Remember there are no right or wrong answers, and what is said in this group stays confidential (within the group).

1. Let us discuss what you know about the relationship between TB and alcohol use in your community.
   Probes: Think about kinds of alcohol use, how much and when people drink alcohol, and who they may use alcohol with? What are the kinds of patterns that are more likely to be associated with TB in people that you know?
   From what you have seen in the community, are people who drink more likely to develop TB? What do you think are the reasons for this?
   What are some of the reasons that people who have been diagnosed with TB may use alcohol?

   2. When people go on TB medication, are they spoken to about alcohol use and told about how it can affect their TB by staff in the clinic? *Probe:* Are there any specific talks or teaching about importance to cut back or need to stop alcohol use for health reasons?

   3. What are some of the other effects that using a lot of alcohol, or drinking often, may have in people who have TB's lives in the area that you live in?
      Probes: Think about other areas of health (other than TB) that may be affected? What about relationships with important people in their lives, such as family, partners and friends?

   4. In your opinion, how do you think alcohol use affects treatment if someone has TB?

Probe: how could it affect taking medication regularly or staying on treatment? Do you think that alcohol use and TB medications interact with each other? What we mean here is does alcohol have any effects on TB meds, or do TB meds have any effects on what happens to people when they drink alcohol?

5. Can you tell us a bit about any changes in alcohol use or drinking- that you think individuals may make once they are diagnosed with TB or have had TB before?
   Probe: What kinds of changes may occur in drinking (types, frequency, amount etc)
   How are these changes made? What kinds of things would help someone make these changes?
   How long do these changes usually last for individuals who have TB? Is this still the case when someone begins to feel better?

6. What could some of the reasons be for someone not changing their drinking behaviours when they are diagnosed with TB?
   Probe: Do you think that some of these people want to change their alcohol use but there are things in the way of making these changes?
   These can refer to individual, interpersonal or structural barriers?

7. From what you know, how do you think TB patients in your community who may struggle with their drinking are identified at the health clinic?
   Probe: Who would usually identify this kind of alcohol use? When would this occur?
   In your opinion, should all patients who present with TB be asked about their alcohol use, or only certain patients? If only certain patients, how would healthcare providers decide which patients to ask about their alcohol use?
   Where else do you think alcohol use is sometimes picked up among TB patients outside of the healthcare clinics? Can you tell me a little bit more about what you know about this?

8. **Now I want to ask you a few more questions about stigma, and how people have been treated that use alcohol and have been diagnosed with TB.**
   a. From what you know and have seen in your community, how are people who use alcohol and have TB treated differently or badly (stigmatized) by healthcare providers (including community health workers) to others?
      i. **Probe:** What do you think are some of the reasons for this stigma (e.g., substance use, TB or other issues such as mental health, HIV, poverty)?
      ii. Can you tell us about any differences in how these individuals have been treated since the COVID-19 pandemic?

        iii. How would this kind of stigma impact people and their access to health care (for TB and/or alcohol use)?

   b. Has this stigma also been expressed or exist from other people (friends, family, or in the community in general) for people who use alcohol and TB?
        i. Probe: have you heard about people in your community who have experienced, felt, or noticed any stigma around this?
        ii. Probe: What do you think this is like if the person has TB as well as COVID-19?
        iii. Do you have any ideas about what would need to happen for some of this stigma (negative thoughts and behaviours) towards this group of people to be changed in the community that you live in?

9. Do you know of people like you in the community, who were being treated for TB and have problems with their drinking, who have been told about or sent to services from the health care clinic because of their alcohol use?
   a. *Probe*: What do you know about how this would work? Do you think other people in the community of aware of these services?
   b. What have you heard about the types of services that they were sent on for?
   c. Would you go to alcohol services outside of the healthcare setting/clinic (like rehab, treatment by a community organization or religious orgnisation)? Why/why not?
   d. What are some of the things that would be important for health care providers like nurses or doctors to think about when telling people who they know have problems with their drinking and have TB when they send them to other services/treatment? What would influence whether they attend these services or not (e.g. access to these services, costs etc)?
   e. What about during COVID-19, do you think services/treatment for people who used alcohol and had TB in your community were affected and how?

Thank participants for their time and remind them that everything that was mentioned in the group will be kept confidential.
