# Supplementary material for: Patient and provider perceptions of the relationship between alcohol use and TB and readiness for treatment: a qualitative study in South Africa
Source: BMC Public Health. 2024 Aug 14;24:2216. doi: 10.1186/s12889-024-19570-y (PMC11325746; doi:10.1186/s12889-024-19570-y)
Supplement: Supplementary file 2 — Supplementary Material 2 [file 12889_2024_19570_MOESM2_ESM.pdf]

Key Informant Interview Guide

Read: Thank you for agreeing to this interview today. The information that we gain from speaking with you today, as well as with other experts working with individuals diagnosed with TB and who use alcohol, will inform us about available services in the community as well as what additional needs individuals with TB may have, and what this has been like during COVID-19. As a reminder, we will be audio-taping this interview, however your individual responses will remain confidential and will not be linked to you or the organization that you work for.

1. From your experience, tell us a bit more about the use of alcohol among individuals who are diagnosed with TB and what you think the relationship between alcohol use and TB is?
    a. Probe:
        a. In your experience how would you describe the patterns of alcohol use that may increase risk for TB? (type of alcohol, frequency, quantity)
        b. What kinds of (if any) risks/consequences for TB-positive individuals who drink alcohol have you seen?
        c. How (if relevant) does the work that you do address alcohol use among individuals who have been diagnosed with TB?

2. How much screening for alcohol use disorders currently happens within TB services at health care clinics? How about at the TB hospital?
        a. Probe: is alcohol use routinely asked about? how would it normally be detected?
        b. Can you tell us about any potential benefits to routinely screening TB patients for alcohol use?
        c. What may be some of the challenges in routinely assessing individuals who present with TB for alcohol use?
        d. What do you know about screening tools that you may have personally used, or you have heard of others using, to detect alcohol use in TB services within the healthcare setting?

3. What are the kinds of roles that other members of the TB clinical team can play in t providing services for people diagnosed with TB that may have problem alcohol use? Let’s discuss these services a bit.
    a. Probe: Tell us more about the kind of information that may be provided around alcohol use for TB patients in healthcare settings. Who provides this information-is it nurses or also others?
    b. What are your thoughts about DOTS workers discussing alcohol use with patients, especially around taking TB medication? Any suggestions about how this might work?

c. What other information for problem alcohol use do you think may be provided by other health care workers?

4. We are also thinking about the services that patients with TB, who have problems with their alcohol use, may need within the healthcare setting.
    a. What, if any, kinds of intervention or treatment services (psychological or pharmacological) are available within the healthcare setting for individuals who use alcohol?
    b. What are the gaps in existing services that need to be addressed?
    c. How ready to receive services/treatment do you think participants would be, based on your experience? What are some of the reasons for this?
    d. What would some core elements of such a programme be that would be beneficial to this group of people who use alcohol with TB (delivery, timing in terms of diagnosis etc)?
    e. What has the feedback been from patients with TB who use alcohol about the COVID-19 vaccine in terms of knowledge of uptake? Do you think that there are any specific barriers that this group may face in receiving the vaccine?

5. We are also interested in other services outside of the healthcare setting that people who use alcohol and have TB may need.
    a. What other services do you think that people who have problem drinking may need when on treatment for TB? Who do you think would be best placed to help work with this group of people?
    b. Can you tell us a bit more about the services that were available in the community for people who use alcohol and have TB prior to COVID-19? Has this changed during COVID-19?
    c. What has the feedback been from patients with TB and who use alcohol- about the COVID-19 vaccine in terms of knowledge of uptake? Do you think that there are any specific barriers that this group may face in receiving the vaccine?

6. What do you know about referral services that are available for problem alcohol use among people with TB who may need more help for their alcohol use?
        a. Probe: Are there referral networks between services for alcohol and TB? If yes, how often do people go for alcohol services if referred?
        b. In your opinion, how well do these referral pathways work?
    b. If referrals are made for people with TB who have problem alcohol use, what do you think could be some of the reasons for people with TB and alcohol use disorders not taking up these referrals (barriers such as readiness to change)?
    c. What kinds of suggestions do you have about how to address some of these barriers?

7. Based on your experience, tell us about the kinds of stigma that people that have TB and problem alcohol use may experience if they do go for healthcare.
   Probe. a. How could this affect their ability to receive services for alcohol use?

b. What kinds of stigma may these patients who have been diagnosed with COVID-19 face?
c. Any thoughts about how this could be addressed within the healthcare setting?

8. We would like to hear your thoughts about how to improve screening and referral systems for problem alcohol use within the healthcare system.

a. Probe: If screening for alcohol were provided uniformly, when and how often during services do you think patients should be assessed for alcohol use?
b. What, in your opinion, would a feasible and acceptable screening tool for alcohol use look like?
b. Who at the healthcare clinic could be responsible for the assessment/detection of alcohol use?
c. What do you think should be the next step if someone is screened, and it appears their alcohol use may be a problem? When would referrals to outside organisations be suggested or when could the clinic help the patient with their alcohol use?
d. Can you tell us what you think a good referral pathway for alcohol services would look like?

Thank them for their time, and ask if they have any additional questions.
